# Estimating Genome-Wide Phylogenies Using Probabilistic Topic Modeling

**DOI:** 10.1093/sysbio/syaf015

**Published:** 2025-02-25

**Authors:** Marzieh Khodaei, Scott V Edwards, Peter Beerli

**Affiliations:** Department of Scientific Computing, Florida State University, 110 N Woodward Ave, Tallahassee, FL 32306, USA; Department of Organismic and Evolutionary Biology and Museum of Comparative Zoology, Harvard University, 26 Oxford Street, Cambridge, MA 02138, USA; Department of Scientific Computing, Florida State University, 110 N Woodward Ave, Tallahassee, FL 32306, USA

**Keywords:** Alignment-free, bootstrap, CONTML, *k*-mers, LDA, multilocus phylogeny, NLP, topic modelling

## Abstract

Methods for rapidly inferring the evolutionary history of species or populations with genome-wide data are progressing, but computational constraints still limit our abilities in this area. We developed an alignment-free method to infer genome-wide phylogenies and implemented it in the Python package TopicContml. The method uses probabilistic topic modeling (specifically, Latent Dirichlet Allocation) to extract "topic" frequencies from k-mers, which are derived from multilocus DNA sequences. These extracted frequencies then serve as an input for the program Contml in the PHYLIP package, which is used to generate a species tree. We evaluated the performance of TopicContml on simulated datasets with gaps and three biological datasets: 1) 14 DNA sequence loci from two Australian bird species distributed across nine populations, 2) 5162 loci from 80 mammal species, and 3) raw, unaligned, nonorthologous PacBio sequences from 12 bird species. We also assessed the uncertainty of the estimated relationships among clades using a bootstrap procedure. Our empirical results and simulated data suggest that our method is efficient and statistically robust.

Phylogenetic analysis traditionally relies on the alignment of orthologous sequence data, a process that can be challenging due to the complexity of genomic variations, difficulties in aligning noncoding regions, and the presence of highly divergent sequences. Over the past two decades, alignment-free approaches based on shared properties of subsequences of defined length k (k-mers or k-grams) (Deschavanne et al., 1999; Chapus et al., 2005; Shedlock et al., 2007; Marçais and Kingsford, 2011) have been developed to compare sequences and genomes. Alignment-free methods for evolutionary analysis have been reviewed (Vinga and Almeida, 2003; Zielezinski et al., 2019) and their robustness investigated (Chan et al., 2014; Bernard et al., 2016). For example, they can be used to derive distances to be summarized into phylogenies (Edwards et al., 2002; Chan et al., 2014; Balaban et al., 2022; Van Etten et al., 2023). Several studies have shown that alignment artifacts can significantly impact tree topology (Ogden and Rosenberg, 2006; Wong et al., 2008; Du et al., 2019). Alignment becomes problematic with comparisons of large genomes, complex genomic variations, challenges in aligning noncoding regions, difficulties presented by highly divergent sequences, and the time-consuming nature of aligning large datasets. Alignment-free approaches offer a promising alternative to address these weaknesses of alignment-based methods (Ren et al., 2018).

Probabilistic topic modeling (Blei et al., 2003) is a statistical approach aiming to identify major "themes," "connections," or "topics" among themes in documents and other large collections of text. The approach originated from the field of Natural Language Processing (NLP) and was introduced by statisticians looking for applications of machine learning. Griffiths and Steyvers (2004) applied this method to infer a natural grouping (topics) of documents based on the content from a large number of scientific documents, called a corpus. Latent Dirichlet Allocation (LDA) is a popular technique in topic modeling within the context of unsupervised machine learning, introduced by Blei et al. (2003). The goal is to uncover these topics by analyzing the words in the documents and essentially "learning" the structure of the data. The method assumes that documents consist of latent topics, each represented by a distribution of words. LDA has also been a focus of attention within the bioinformatics community and various applications to biological data have been researched and analyzed (Liu et al., 2016). Recently, some applications of alignment-free methods have been presented to solve problems offered by DNA sequences or genomes. For example, in a statistical application, LDA was used by La Rosa et al. (2015) to extract the frequency of fixed-length k-mers (words) of DNA sequences (documents) and thereby discover latent patterns in massive biological data to be used for clustering and classification of DNA sequences. Other studies have adopted LDA clustering for the analysis of single-cell expression or epigenetic data (duVerle et al., 2016; Dey et al., 2017). Here, we present a novel computational approach using probabilistic topic modeling to infer evolutionary relationships among individuals from different populations or species. This method works with multilocus data, including unaligned or aligned DNA sequences and unassembled raw sequencing reads. We will use the term species tree for these trees, whether derived from single individual sequences or individuals grouped into populations or species.

Genome-wide datasets (sequences of whole genomes or multiple genes per species) are becoming increasingly prominent in inferring the evolutionary history of closely related species. Traditional approaches to multilocus phylogenetics, such as concatenation methods (Gatesy and Baker, 2005), and approaches that are consistent under the multispecies coalescent (de Queiroz and Gatesy, 2007; Liu et al., 2009; Chifman and Kubatko, 2014; Zhang et al., 2018; Mirarab et al., 2016), have advanced the field significantly. However, these methods often rely on high-quality alignments, which can be computationally expensive and error-prone when dealing with large or complex datasets. TopicContml offers a scalable and efficient alternative to traditional approaches, addressing their limitations and enabling the analysis of diverse and complex genomic datasets without relying on sequence alignment.

Here, we outline the architecture of TopicContml and demonstrate its application using simulated data and three empirical datasets: 1) a small orthologous dataset of individuals from various locations of two parapatric bird species, consisting of 14 loci, along with a comparison to SVDquartets (Chifman and Kubatko, 2014) and the alignment-free method Mash (Ondov et al., 2016), including a discussion of bootstrap support; 2) an orthologous dataset of mammalian species, consisting of 5162 loci across 90 vertebrate species; and 3) a 12-species bird dataset with unaligned, nonorthologous PacBio raw sequencing reads.

## Materials and Methods

### TopicContml Software

**Fig 1:**
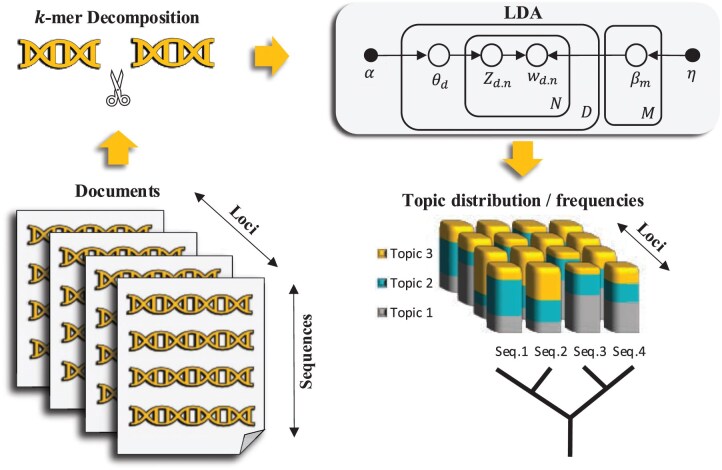
TopicContml workflow to generate topic frequencies and the corresponding phylogeny.

TopicContml is a Python package based on a two-phase pipeline: 1) The mulitlocus or genome-wide sequences are fragmented into k-mers; these k-mers are then used to learn a probabilistic topic model and extract the topic frequencies of these k-mers using the LDA model for each locus (Fig. 1 left). For a data analysis with multiple individuals from the same species or population, we set the option--merging n to merge individual labels that start with the same n letters into groups; otherwise, we assume that each sequence is an individual. 2) These topic frequencies from multiple loci are then used to estimate a phylogeny with Continuous Characters Maximum Likelihood (Contml) (part of PHYLIP; Felsenstein, 1981, 2004) (Fig. 1 right).

#### K -merdecomposition

In NLP, large text datasets (corpora) are broken down into smaller units such as documents, which are further divided into words or sentences, referred to as tokens. Similarly, in bioinformatics, datasets consisting of multiple genomes or multilocus DNA sequences can be broken down into individual genomes or groups of multilocus sequences associated with an individual. These sequences are then decomposed into k-mers—substrings of length k representing short DNA or amino acid sequences. We decompose the DNA sequences into nonoverlapping k-mers, as shown in Figure 1, since overlapping k-mers require more memory and computation time without yielding significantly better results. The program estimates an optimal k-mer length based on the probability of observing a given k-mer in a document (sequence) at each locus, with options for user adjustments.

The probability of a given k-mer K appearing in a random genome X of size n is $P(K\in X)=1-{\left(1-|\Sigma {|}^{-k}\right)}^{n}$, where Σ={A,C,G,T}, without loss of generality. Given a document size n and the desired probability q of observing a random k-mer, the value of k that minimizes the probability of observing a random k-mer can be computed as (Fofanov et al., 2004; Ondov et al., 2016)


$$\hat{k}=\lceil log_{\left|\Sigma \right|}(n(1-q)∕q)\rceil .$$

TopicContml calculates $\hat{k}$ for each document in a locus based on this. We have found that k=20 and k=8 give accurate estimates in most cases for large (e.g., 1,000,000 bp) and small sequences, respectively. The program also allows users to choose different k-mer configurations, such as a combination of k-mer lengths or a single fixed length, based on their analysis needs.

#### Resolving ambiguities and missing data

The current version of TopicContml retains all IUPAC codes as they are, except for “N,” “?,” “-,” and other ambiguous characters, which can be filtered out prior to analysis. We assume that such ambiguity codes are rare and do not strongly affect the results.

#### Topic modeling

Given a collection of D documents and a number of M topics, topic modeling discovers the M topics from a collection of text data and estimates the probability of each topic for each document. We use LDA to extract these frequencies. LDA is a generative probabilistic model used to uncover hidden topics within a collection of documents, referred to as a corpus. Given a corpus with *D* documents, let N be the number of words in a specific document d∈D. For each word $w_{d,n}$ (the nth word in the dth document), $z_{d,n}$ denotes the associated topic. The distribution of topics for document d, represented by $\theta_{d}$, is drawn from a Dirichlet distribution, $\theta_{d}∼Dir(\alpha )$, where α>0 is the parameter vector. Similarly, the distribution of words for each topic m, denoted by $\beta_{m}$, is also drawn from a Dirichlet distribution, $\beta_{m}∼Dir(\eta )$, with parameter vector η>0. In this model, the only observed variables are the words w in the documents, while the topics z, the topic distributions θ for all documents, and the word distributions β for each topic are all latent variables. The joint distribution is defined as


$$\begin{array}{llll} & p(w,z,\theta ,\beta |\alpha ,\eta ) & & \\ & =\underset{m}{\prod }p(\beta_{m}|\eta )\underset{d}{\prod }\left[p(\theta_{d}|\alpha )\underset{n}{\prod }p(z_{d,n}|\theta_{d})p(w_{d,n}|z_{d,n},\beta )\right]. & & \end{array}$$

Using this joint distribution, one can compute the posterior distribution of the unknown model parameters, $p(\theta ,z,\beta |w)=\frac{p(\theta ,z,\beta ,w)}{p(w)}$, using expectation-propagation (Minka and Lafferty, 2012) or other maximization methods.

For each locus, TopicContml first estimates the topic frequencies for every document, θ, using the Python package Gensim (Řehůřek and Sojka, 2010) (Fig. 1). The sequences (documents) in each locus are decomposed into k-mers (words). During preprocessing, LDA filters out certain words, primarily those with low frequency, to improve topic coherence and reduce noise in the learned distributions (see Supplementary Fig. S8). The process begins by randomly assigning a distribution of topics to each document and a distribution of words to each topic, with these distributions being governed by Dirichlet priors. During the training phase, Gensim’s implementation of LDA iteratively refines these distributions using maximization methods. This training involves updating two key parameters: the distribution of topics within each document and the distribution of words within each topic. The model trains by analyzing patterns of word co-occurrence across the documents, assigning words to topics in a way that maximizes the likelihood of the observed data. As the iterations progress, the model converges to a stable set of topics.

#### Determining the optimal number of topics

Selecting the optimal number of topics in LDA modeling is important for generating interpretable results. A widely used method for this involves evaluating topic coherence, which reflects the semantic similarity among top words within each topic—higher coherence scores generally correlate with more interpretable topics (Röder et al., 2015). Several algorithms are available for calculating coherence scores. In this study, we use the “$u_{\mathrm{mass}}$" coherence measure in Gensim (Řehůřek and Sojka, 2010), analyzing each locus individually to identify the best topic number for each (see Supplementary Fig. S3). However, because coherence analysis involves testing multiple models with varying topic counts, it can be time-intensive, especially with large datasets. To address this, TopicContml also allows users to specify a fixed number of topics, which, in our case, proved to be a practical alternative without compromising the interpretability or consistency of results. Although selecting an optimal topic count can enhance detail in some studies, our findings show that a fixed topic number performs well and offers efficiency for large-scale analyses. All our analyzed datasets are based on a fixed value of five topics.

#### Visualizing topics and associated terms

To visualize the topics and associated terms (k-mers), we use the package pyLDAvis (Sievert and Shirley, 2014), an interactive visualization tool. TopicContml allows users to generate an HTML file containing the pyLDAvis output for each locus, saving them in a designated folder within the directory (see Supplementary Fig. S2). This enables detailed examination and extraction of information from the fitted LDA model, enhancing our ability to interpret the underlying topic structures.

#### Contml (Continuous Characters Maximum Likelihoodmethod)

The results of the LDA analysis are the topic frequencies for each document, which are then evaluated using Contml to estimate a phylogeny from frequency data using the restricted maximum likelihood method (Felsenstein, 1973) based on the Brownian motion model for allele frequencies (Cavalli-Sforza and Edwards, 1967). The primary assumption of Contml is that each character (each topic in our case) evolves independently according to a Brownian motion process and that character state changes since divergence are independent of each other, which means that the net change after t units of time is normally distributed with zero mean and variance ct (the same constant c for all characters).

#### Multilocus bootstrapping

To assess the statistical confidence of the inferred phylogenies, we use bootstrapping (Efron, 1979). Given a multiple sequence alignment, the bootstrap method involves resampling the original dataset with the replacement of the aligned sites and creating the phylogeny for each replicate. For unaligned data, we use the approach used in Edwards et al. (2002), where a random sample of x  k-mers is drawn from all x  k-mers collected from the data. The fraction of the time a particular clade appears in the resulting bootstrap trees presents support values for the clades in the reference tree or a majority-rule consensus tree (Hillis and Bull, 1993; Efron et al., 1996; Holder et al., 2008). TopicContml implements bootstrapping strategies for both aligned and unaligned sequences. It generates a majority-rule consensus tree from the bootstrap replicates using SumTrees in DendroPy (Sukumaran and Holder, 2010).

### Datasets

We evaluate the accuracy of TopicContml for simulated data and multiple real biological data. The simulated data were used to explore the effects of the number of loci and the accuracy of recovering the true topology from data sets consisting of 7 and 14 species. We used three biological datasets with very different features: 1) a 14-locus dataset from 2 parapatric closely related bird species separated into 9 populations, to evaluate the accuracy of estimation, bootstrapping, and to compare accuracy with SVDquartets (Chifman and Kubatko, 2014) and the alignment-free approach Mash (Ondov et al., 2016); 2) a vertebrate dataset focusing on mammals with 90 species and 5162 loci to evaluate the effect of missing data and of aligned versus nonaligned orthologous loci; 3) a dataset of raw PacBio sequences of 12 bird species, each containing 100,000 reads; these sequences were neither orthologous nor aligned and can be construed as having been sampled randomly and potentially overlapping from their constituent genomes.

#### Simulated datasets

**Fig 2:**
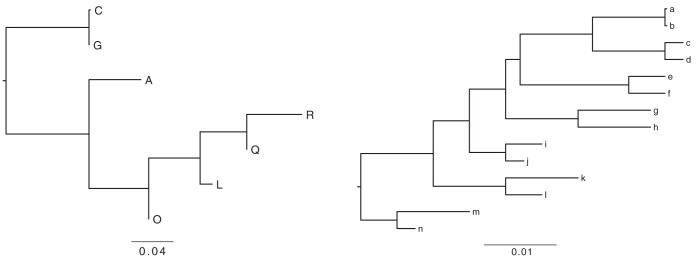
Phylogenies of 7 and 14 species used for the simulation of sequence data with deletions and indels (gaps). In Tables 1 and 2, these are referenced as 7-tip and 14-tip trees.

We evaluated two sets of simulations: one with moderate and one with many insertions and deletions. We used the software Dawg (Cartwright, 2005) to simulate aligned sequences with indels/deletions on a 7-species and a 14-species tree (Fig. 2). The moderate scenario inserts indels at a rate of 0.02 per site and deletes sites with a rate of 0.02 per site. The more extreme simulation used an insertion/deletion rate of 0.2 per site. An example of two individual sequences for each indel/deletion scenario is shown in the electronic supplement (Supplementary Fig. S1). We simulated datasets of 1, 2, 5, 10, 20, 50, 100, 200, 500, and 1000 loci; each locus was between 800 and 2000 bp, dependent on indels/deletions. These simulated datasets were then analyzed with gaps included (aligned), with the gaps excised (unaligned), and with k-mers that include gaps removed (no gap-kmer). We fixed the number of topics for all simulation analyses to 5 and estimated the best k-mer size from the data; it turned to be 9 for all datasets. Each scenario was run 100 times. The resulting trees were compared with the true trees using unweighted and weighted Robinson–Foulds (RF) distances.

#### Empirical datasets

We evaluated three biological datasets:

##### Two parapatric closely related bird species:

The data contain multiple individuals of the Australian brown tree- creeper (*Climacteris picumnus*) and black-tailed tree creeper (*Climacteris melanurus*) (Edwards et al., 2022, 2023). DNA sequences consisted of 14 loci and 9 different geographic locations. For each locus, sequence length varied from 288 to 418 base pairs, and the number of aligned sequences per locus ranged from 78 to 92. For the evaluations of unaligned data, we removed all gaps in each sequence. We applied LDA to each locus across all nine locations and extracted the topic frequencies. These topic frequencies were used in Contml to generate the population tree, evaluate bootstrap support, and compare with another approach.

##### Mammal dataset:

The second dataset includes 90 vertebrate species focusing on mammals with 5162 loci (Wu et al., 2018) and was analyzed by Liu et al. (2017) using concatenated maximum likelihood and coalescent approaches. We analyzed this dataset using TopicContml under four conditions: 1) excluding k-mers containing gaps ("-") or unspecified nucleotides ("N"), 2) removing alignment columns with gaps before excluding k-mers with "N," 3) removing all gaps from sequences before excluding k-mers with "N," and 4) using aligned sequences while retaining "N" characters. We then used the RF distance to compare each phylogram with the maximum-likelihood tree and random trees.

##### PacBio dataset:

A total of 6.5 GB of raw sequencing reads of 12 bird species generated by the PacBio HiFi sequencing method. The 12 birds cover most of the depth of the avian tree, including the two deepest branches; Paleognathae and Neognathae Species were chosen so as to broadly sample the tree for species belonging to lineages whose higher relationships are fairly stable, but also to include unambiguously close relatives, so that we could test the ability of our method to recover close relatives (Jarvis et al., 2014; Oliveros et al., 2019). We subsampled 100,000 reads from each species; average read length per species varied from 9.3 kb in the tinamou Crypterellus tataupa to 18.8 kb in chicken.

## Results

### Analysis of Simulated Datasets

The simulations based on the moderate insertion/ deletion scheme, shown in Table 1, demonstrate that the recovery of trees close to the true tree improves with the number of loci for all treatments and also for both tree topologies. The "Aligned" treatment for both tree topologies works well with more than 50 loci. The "Not aligned" treatment worked better for the 14-species tree than for the 7 species tree, but we used only a small number of replicates (n=100). Still, accurate recovery of the true tree was almost as high as with the "Aligned" treatment. The "No gap-kmer" treatment fairs as well as the "Not aligned" treatment. Weighted RF distances (wRF) show trends consistent with the percentage of trees recovered that either match the true tree or are within two distance units of it. Notably, the ’No gap-kmer’ treatment is closer to the true tree than the ’Not aligned’ treatment, which performs comparably to the ’Aligned’ treatment.

**Table 1:** Simulated data with low indel/deletion frequency and accuracy of phylogenetic reconstruction with TopicContml: data were simulated using Dawg (Cartwright, 2005), each locus has around 1000 bp with gaps using a low number of gap scenario (parameter: insertion/deletion probability 0.02/site, average indel length 12, topics=5, k-mers were estimated from the data (all estimated k-mers were 9 base pairs); ’Aligned’ results are based on simulated data with gaps, ’Not Aligned’ had all gaps removed in the simulated data, "No gap-kmer" had all k-mers containing gaps removed before LDA. "C" (Close) marks the frequency of topologies that are either the same as the true topology or not more than 2 rearrangments apart; "wRF" is the average weighted RF distance from the true tree. Each result is based on 100 simulations.

Loci	7 Species						14 Species					
	Aligned		Not Aligned		No gap-kmer		Aligned		Not Aligned		No gap-kmer	
	C	wRF	C	wRF	C	wRF	C	wRF	C	wRF	C	wRF
1	0.33	1.30	0.27	1.26	0.39	1.30	0.00	1.84	0.00	1.68	0.01	1.81
2	0.61	1.21	0.35	1.25	0.54	1.22	0.02	2.01	0.00	2.07	0.03	1.95
5	0.64	1.20	0.53	1.22	0.64	1.20	0.24	2.01	0.13	2.17	0.27	1.96
10	0.70	1.18	0.47	1.19	0.69	1.18	0.35	2.03	0.31	2.14	0.40	2.00
20	0.78	1.17	0.67	1.20	0.78	1.18	0.64	2.00	0.74	2.14	0.58	2.00
50	0.85	1.17	0.76	1.18	0.70	1.19	0.83	2.01	0.90	2.14	0.81	1.99
100	0.93	1.17	0.74	1.20	0.87	1.19	0.93	1.98	0.96	2.14	0.92	1.97
200	0.97	1.17	0.87	1.19	0.95	1.17	0.95	1.98	1.00	2.13	0.96	1.97
500	0.99	1.17	0.88	1.20	0.99	1.17	0.99	1.97	1.00	2.13	1.00	1.96
1000	1.00	1.17	0.94	1.19	1.00	1.18	1.00	1.97	1.00	2.13	1.00	1.95

The simulations with extreme insertion/deletion strategy (as shown in Table 2) are markedly different for the ’Not Aligned’ treatment of the 14 tip trees: even with 1000 loci none of the estimated trees were close to the true tree; ’Aligned’ and ’No gap-kmer’ faired similar to the moderate indel/deletion scheme. For the 7-species tree all treatments are close to the true tree when the number of loci is large. Overall the wRF values are all higher than those for the moderate indel/deletion scenario, and the "No gap-kmer" delivers similar values as the "Aligned."

**Table 2:** Simulated data with high indel/deletion frequency and accuracy of phylogenetic reconstruction with TopicContml: data were simulated using Dawg (Cartwright, 2005), each locus has around 1000 bp with gaps using a high number of gap scenario (parameter: insertion/deletion probability 0.2/site, average indel length 12, topics=5; k-mers were estimated from the data (all estimated k-mers were 9 base pairs); "Aligned" results are based on simulated data with gaps, "Not Aligned" had all gaps removed in the simulated data, "No gap-kmer" had all k-mers containing gaps removed before LDA. "C" (Close) marks the frequency of topologies that are either the same as the true topology or not more than 2 rearrangments apart; "wRF" is the average weighted RF distance from the true tree. Each result is based on 100 simulations.

Loci	7 Species						14 Species					
	Aligned		Not Aligned		No gap-kmer		Aligned		Not Aligned		No gap-kmer	
	C	wRF	C	wRF	C	wRF	C	wRF	C	wRF	C	wRF
1	0.40	1.25	0.18	1.30	0.22	1.28	0.00	1.86	0.00	2.06	0.00	1.85
2	0.47	1.17	0.23	1.22	0.48	1.22	0.07	2.03	0.00	2.96	0.03	1.98
5	0.60	1.19	0.30	1.23	0.56	1.18	0.16	2.08	0.00	3.31	0.17	2.00
10	0.61	1.19	0.42	1.21	0.57	1.20	0.29	2.09	0.00	3.65	0.36	2.03
20	0.62	1.20	0.57	1.23	0.57	1.22	0.58	2.11	0.00	3.70	0.63	2.02
50	0.59	1.22	0.75	1.20	0.45	1.22	0.84	2.10	0.02	3.78	0.75	2.02
100	0.66	1.21	0.76	1.21	0.58	1.22	0.93	2.09	0.01	3.78	0.93	2.00
200	0.74	1.22	0.86	1.21	0.56	1.23	0.97	2.07	0.02	3.82	0.97	1.99
500	0.88	1.20	0.93	1.22	0.55	1.22	0.99	2.06	0.01	3.83	1.00	1.98
1000	0.82	1.21	1.00	1.22	0.59	1.22	1.00	2.05	0.02	3.81	1.00	1.98

### Analysis of Empirical Datasets

#### Multilocus species tree from closely related Australian birds

**Fig 3:**
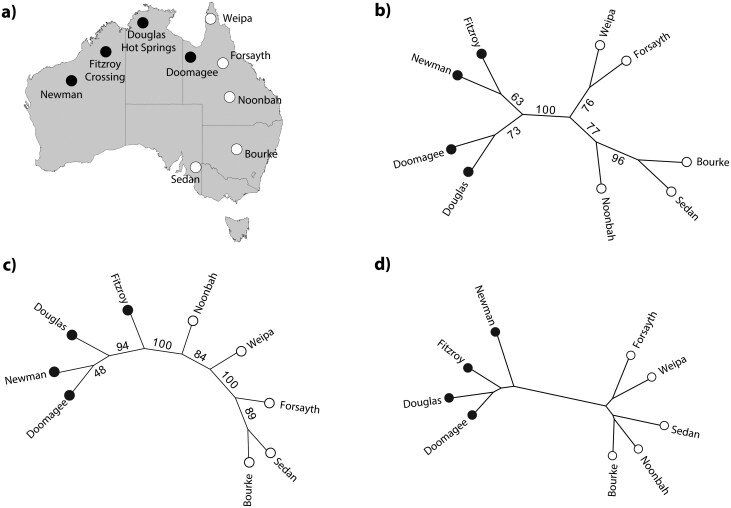
Relationship tree of nine populations of two Australian treecreeper species reconstructed. (a) The map of Australia shows the locations; the black disks mark *Climacteris melanurus*, and the white disks mark *Climacteris picumnus* (Edwards et al., 2023). (b) The majority-rule consensus tree of unaligned data by TopicContml. For each bootstrap analysis, 1000 replicates were used. Values in the graphs are % support. (c) The majority-consensus tree of aligned data analyzed by SVDquartets  +Paup*. (d) The phylogeny constructed by Mash.

For the treecreeper dataset, we tokenized each DNA sequence at every locus using k-mer representation with a k value of 8 (as estimated by TopicContml), employing nonoverlapping tokens. Individuals from the same location were merged, and LDA was applied to the corpus to generate topic frequencies for each locus for each of the nine populations. We used five topics in our analysis. These multilocus topic frequencies were then used to construct a maximum-likelihood tree with Contml in the PHYLIP package. We did bootstrapping, and Figure 3b shows the majority-rule consensus tree generated by TopicContml for unaligned data, with bootstrap support values derived from 1000 replicates. Inspection of the clades reveals that our tree recovers the expected geographic relationship within each species, and the locations are separated by species, which in turn are separated by the Carpentarian barrier in Australia (Cracraft, 1986; Edwards et al., 2023).

We compared the performance of TopicContml with SVDquartets (Chifman and Kubatko, 2014) implemented in Paup* (Swofford, 2003) (SVDquartets  +Paup*), as shown in Figure 3c. There are notable differences between the results of SVDquartets and TopicContml. When comparing these results to the mapped locations in Figure 3a, we observe that our bootstrap tree from TopicContml recovers the relationships equally well or better than SVDquartets. Both methods encounter challenges in resolving certain population splits but confidently separate the two species. TopicContml support values recover, in general, the geographic pattern of the locations well.

We also compared our phylogenetic tree with one generated using the alignment-free approach Mash (Ondov et al., 2016), which estimates evolutionary distances between nucleotide sequences. For the Mash input, sequences from different loci for each individual were concatenated, with missing data filled by gaps. The sequences from individuals at the same location were then merged to create nine Fasta files, representing the nine populations in Australia. The pairwise distance matrix generated by Mash was used to construct a Neighbor-Joining tree using the PHYLIP package (Felsenstein, 2004). Figure 3d shows the phylogenetic tree generated by Mash. When comparing our tree to the one generated by Mash, we observe that both trees depict similar relationships among species.

#### Multilocus species tree from genome-wide mammal dataset

**Fig 4:**
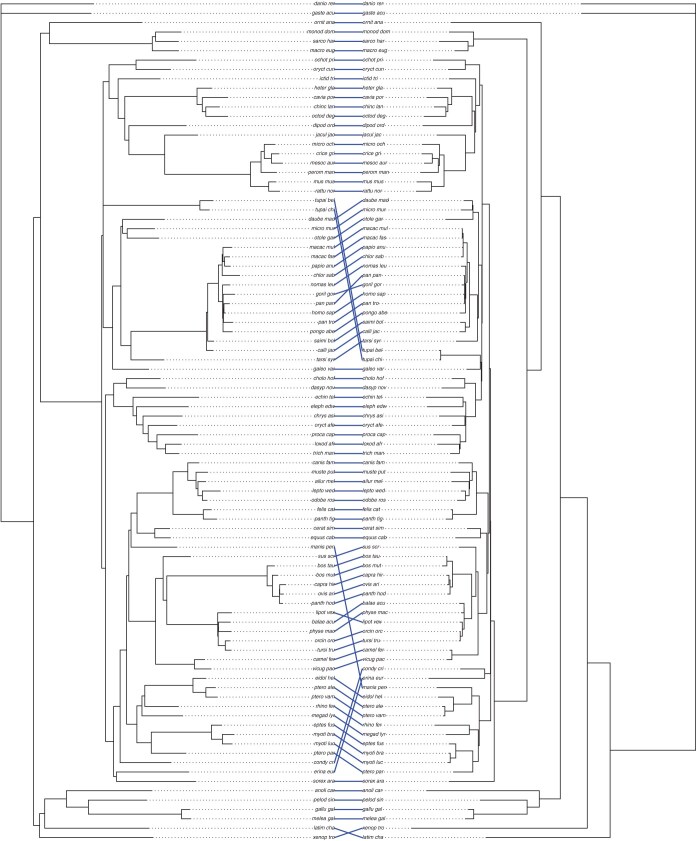
Tanglegram of mammal dataset comparing the TopicContml tree (left), generated by excluding k-mers containing "-" or "*N*," with the maximum likelihood tree from Liu et al. (2017) (right). The alphabetical list of the species names in the tree is in Supplementary Table S1.

We analyzed a mammal data with 90 species and 5162 loci . The dataset consisted of nucleotide characters from the set "ACGT-N." We analyzed the mammal dataset under various conditions and compared the resulting trees to the maximum likelihood tree derived from 4388 loci of 90 vertebrate species, as reported by Liu et al. (2017). The comparisons were visualized using tanglegrams (Revell, 2024). In the first analysis, after generating k-mers, we excluded any k-mers containing either "-" or "N." The resulting phylogenetic tree (Fig. 4) was 60 steps away from the maximum likelihood tree, as measured by the RF distance. In the second analysis, we removed all alignment columns containing gaps, then excluded k-mers with "N." The resulting tree (Supplementary Fig. S4) was 64 steps from the reference tree. This resulted in a tree generated using 1719 loci because 3443 of the 5162 loci did not contain any data after the removal. In the third analysis, we removed all gaps from each sequence before excluding k-mers containing "N." This approach did not improve the tree (Supplementary Fig. S5), which was 90 steps from the reference tree. Finally, in the fourth analysis, we generated a tree using aligned sequences while retaining "N" characters. This tree (Supplementary Fig. S6) was 80 steps from the maximum likelihood tree.

The RF distances between 1,000,000 random trees and the maximum likelihood tree reveal a minimum distance of 168, a mean distance of 173.5, and a maximal distance of 174. The topic modeling trees are therefore considerably closer to the maximum likelihood tree than random trees (Supplementary Fig. S7), confirming that our topic modeling approach recovers phylogenetic signal. The tanglegrams (Fig. 4; Supplementary Figs. S4–S6) also confirm that our tree and the maximum likelihood tree are fairly similar despite a seemingly large RF distance, especially when considering that many of the branches in the mammal tree are very short (Foley et al., 2023).

#### Multilocus species tree from raw unassembled *PacBio* sequence reads

**Fig 5:**
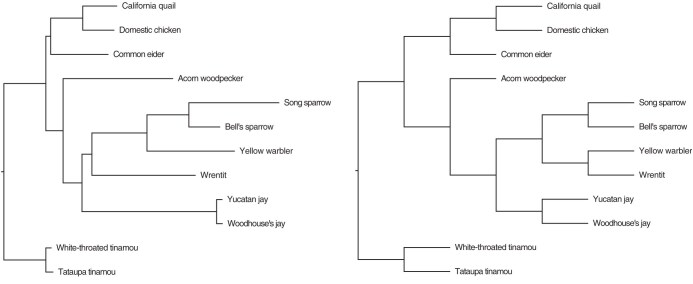
The phylogeny generated by TopicContml (left side) compared with the reference tree (right side).

This dataset consists of FASTA files containing 100,000 reads from each of 12 species. PacBio reads were counted and parsed with Seqkit stats (Shen et al., 2016) and seqtk sample (Li, 2013). To optimize computational efficiency, we concatenated every 1000 reads into single sequences, resulting in 100 loci per species. This approach balances the need to manage sequence length for LDA while reducing the total number of loci, thereby enhancing computational performance. Figure 5 (left) displays the tree generated by TopicContml using a nonoverlapping k-mer length of 20, with an RF distance of two from the reference tree, as shown in Figure 5 (right). The reference tree was drawn from Cracraft (1988), Oliveros et al. (2019), and Wu et al. (2024). The discrepancy in relationships of the Wrentit and Yellow warbler is uncertain, because relationships in this portion of the passerine tree are certainly not definitive (Oliveros et al., 2019).

Given the large size of each document, we performed our first analysis using a longer k-mer length (k-mer length of 20 as we discussed in Section K-mer decomposition). To assess the effects of different k-mer ranges, we experimented with lengths from 8 to 42. We found that optimal results were achieved with k-mers in the range of 18–25, as demonstrated in Supplementary Figure S9.

## Discussion

This study integrates k-mers and probabilistic topic modeling to perform phylogenetic analysis on unaligned or aligned multilocus sequence data, as well as on unassembled raw sequencing reads. The Python code TopicContml offers an efficient workflow to reconstruct evolutionary relationships potentially without prior sequence alignment. TopicContml contains a two-phase analysis. First, k-mers are extracted from DNA sequences, and LDA uses these k-mers to establish how probable an individual’s set of k-mers fits an arbitrary number of topics. For each locus and each individual, we generate a vector of assignment frequencies for a predefined set of topics. This step can be parallelized among loci. The LDA runtime depends on the length of the sequences (documents) and the number of loci. In the second step, the topic frequencies are used as input for Contml to construct a phylogenetic tree. The Contml evaluation time is influenced by the number of tips and the number of loci, as the input matrix for Contml is structured as number of tips × (number of loci × (number of topics - 1)). Although the current version of Contml does not support parallelization, implementing this capability could significantly improve its runtime, particularly for analyses involving many species. As shown in Supplementary Table S3, the Australian bird dataset completed in seconds for both LDA and Contml. The mammal dataset, however, took longer for LDA due to its large number of loci (5,162), despite relatively short sequence lengths, and even longer for Contml due to the large input size. For the PacBio dataset, the LDA runtime was extended by the significantly longer sequence lengths, but the Contml step completed within seconds.

The simulated data experiments reflect the influence of gap handling on phylogenetic reconstruction accuracy under different insertion/deletion (indel) rates and tree complexities. The simulation protocol produced aligned data with low and high gap numbers. The aligned data produced the most accurate phylogenetic inference; for both moderate and high indel frequencies. The observed decline in accuracy for the "Not Aligned" group, especially in the high-indel 14-species tree, stems from the loss of indel information and, because gaps are removed entirely, the formation of new k-mer that do not necessarily coincide with the phylogenetic signal. The "Not Aligned" group faired well with the moderate indel scheme because only few k-mers were affected. The "No gap-kmer" group’s results, which approximate those of the "Aligned" group under moderate indel scenarios, suggest that excluding only gap-containing k-mers strikes a balance between noise reduction and information preservation. This selective approach retains enough informative content while minimizing the alignment artifacts that become problematic in high-indel scenarios.

In contrast, the analysis of the real datasets was more complex, as demonstrated using the mammal dataset to evaluate the effect of structuring sequence data into k-mers. The RF distances to the maximum likelihood tree (Liu et al., 2017) reveal that increasing the number of loci does not always improve phylogenetic accuracy. The mammal dataset analysis highlights the robustness of TopicContml in recovering phylogenetic signal under various treatments of gaps and missing data. The most accurate tree (RF distance of 60) was achieved by excluding k-mers containing gaps or "N," underscoring the importance of targeted ambiguity removal. Removing entire alignment columns with gaps reduced the number of loci but still produced a comparable tree (RF distance of 64). Conversely, removing all gaps from sequences resulted in the least accurate tree (RF distance of 90), likely due to the loss of biologically informative gap signals or formation of phylogenetically noninformative k-mers created at the gap boundaries. Retaining "N" characters in aligned sequences yielded an intermediate result (RF distance of 80), showing that while ambiguity introduces noise, key phylogenetic relationships are still preserved. Notably, all TopicContml trees were substantially closer to the reference tree than random trees.

Tree uncertainty is commonly assessed through bootstrap analysis, which poses challenges for unaligned datasets as it requires bootstrapping at the k-mer level rather than the sequence level. For the treecreeper dataset, bootstrap analysis with TopicContml demonstrates its robustness in recovering phylogenetic relationships from unaligned data. The majority-rule consensus tree effectively separates the two species and accurately captures geographic patterns, including the division across the Carpentarian barrier. Compared with the alignment-based SVDquartets, TopicContml achieves equal or better precision in recovering geographic relationships. Furthermore, the comparison with the alignment-free method Mash confirms that TopicContml effectively captures key phylogenetic relationships while working with unaligned data.

Our analyses of the PacBio dataset shows substantial promise deriving phylogenetic signal from unaligned long-read sequences and demonstrates the potential of TopicContml for alignment-free phylogenetic reconstruction. Despite the complexity of raw, unassembled reads, TopicContml produced trees closely matching a reference phylogeny, showing its capacity to infer evolutionary relationships directly from complex, heterogeneous data. An important goal of the future is to determine what components of unaligned genomic data—tranposable elements, satellite sequences, or other common components of genomes—are driving these positive results. Although the LDA step for this dataset required additional processing time due to long-read lengths, Contml efficiently completed tree inference. These results suggest that TopicContml offers a promising approach for handling high-throughput phylogenetic data without requiring sequence alignment or even genome or locus assembly.

TopicContml is modular, and we have begun work to replace Contml with a network-generating package that may improve the analyses of such datasets by incorporating gene flow between species. Currently, for many datasets that do not suffer widespread introgression, TopicContml allows the analysis of many loci from many individuals that can be grouped, for example, into locations or species. We believe that TopicContml will become a valuable addition to the computational toolkit for phylogenetics by constructing evolutionary trees without or with sequence alignment.

## Software Availability

We implemented our method as a free software named TopicContml under the MIT open-source license. The source code and the documentation of TopicContml are available at https://github.com/TaraKhodaei/TopicContml.git

## Supplementary Material

syaf015_suppl_Supplementary_Materials

## Data Availability

**Simulated Data**: Instructions for generating the simulated data are available at https://github.com/pbeerli/simulations_for_topiccontml. **Australian Treecreeper Dataset**: Available through Dryad (Edwards et al., 2022). **Mammal Dataset**: The aligned mammal dataset can be accessed at https://figshare.com/articles/cds_5162_zip/6031190 (Wu et al., 2018). **PacBio Bird Sequences**: The sources of PacBio long-read sequences from birds are detailed in Supplementary Section 4: PacBio Dataset, under Processing of PacBio Reads, and summarized in Supplementary Table S2. Datasets for the two tinamou species are available on Dryad at doi: 10.5061/dryad.73n5tb36r.
