# Supplementary material for: Estimating Genome-Wide Phylogenies Using Probabilistic Topic Modeling
Source: Syst Biol. 2025 Feb 25;74(5):850–61. doi: 10.1093/sysbio/syaf015 (PMC12699994; doi:10.1093/sysbio/syaf015)
Supplement: syaf015_suppl_Supplementary_Materials [file syaf015_suppl_supplementary_materials.pdf]

Supporting Information for:

Estimating Genome-wide Phylogenies Using Probabilistic Topic Modeling

Contents

1 Simulated dataset **2**

2 Bird dataset **3**

3 Mammal dataset **5**

4 PacBio dataset **10**

5 Runtime for different datasets **11**

1 Simulated dataset

We evaluated two sets of simulations: one with moderate and one with many insertions and deletions. We used the software DAWG (Cartwright 2005) to simulate aligned sequences with indels/deletions on a 7-species and a 14-species tree (Fig. 2). The moderate scenario inserts indels at a rate of 0.02 per site and deletes sites with a rate of 0.02 per site; for example, in the 7-species simulations, a species could have at a locus on average 1038 sites that include, on average per species 8.2 gaps with and average length of 12 sites. The more extreme simulation used an insertion/deletion rate of 0.2 per site. The number of gap sites increased for the specific tree considerably: each locus had about 59.3 gaps on average 17 sites long, each species had about 960 A, C, G, T sites, and about 1000 '-' sites. The branch length and topology matter for the gap distribution: the 14-tip tree had a similar distribution of the gaps with the low insertion/deletion parameter as the 7-tip tree, but the high parameter resulted in many gaps but not as extreme as in the 7-tip tree (about 400 '-' sites per species).

```
#Relevant parameters for DAWG
Root.Length = 1000
Subst.Model = "GTR"
Subst.Freqs  = 0.2, 0.3, 0.3, 0.2
Subst.Params = 2.0, 1.0, 3.0, 1.0, 1.0, 1.0
Indel.Model = "GEO"
Indel.Rate = 0.02
Indel.Max = 2000
Indel.Params = 12
#Example result for the first of 7 species: indel/deletion rate=0.02
   7    1038  #number_of_species number_of_sites
A          GGCTGCGTCAGCTAGGCGACGGACTAGCGGGCCTTCACCCATCAAGTATTCGCTATGGCG
           AGGGTCAATCATCCTTAGGCATCGCATCCCACAAAGAGCGCTGTTATGCAGCCGGCAGCC
           ATAGGCCGGGCTGCTGAAGCGGCCCCGCCAAAGTGGCCCTTAGCGTGGGGGAGCGGCCGG
           ATGTGGTGTCTAACCATCGCTGGTCGCAGTCCGATACACCATATAGCGCCATCGATGGTG
           CTTTCTATCGGAGCTCGTTAGCCAAGTGGTCCGGGTAATTCGGGGACACAATGCTCGTAG
           CTCCGGGCAGCCGTGTGCCATGACTAGTCCCGGCAGTCTCGATGTGCACGACCCTTGCGC
           CTGGTTCGCAGTCTTGCCAATGA----TATGTAGCCGGGGAGTGTCGACAGCCCAGACTG
           GAGACCGCCCCCTTGACGCCTGGATGCTGCTCGTAGGTACTTACGTACCGCTCTAGCGGA
           GTAGAGCCCACCCCGGCGGAGCGGCCAGAGCCACCGGCGTCCTTGTGAC-----------
           ------------------TCCTCTGTGCCTATTGCGAGCTACTTGCCGACCTCTCGCAGG
           TGGAGAGCTCACAGCCCAATAGCCCACCGACCCGTTGGGCCTAGCACGATATTTCCGGCC
           CCCAGAACCAATTCTGGAACACTAAGTCACTGAGCGGTTCGCCATGCCCACCTTTGGTGA
           GGCTTTATAGGCCGTCGGGTACGATGGGCGCCGAGAACACTCTCGGTCTTTCACGGAAGC
           ACAGTGCGGCTGTGTCCGGCGAGTGACCCCCCGGGTACCATAGACAC---GCTAGGC---
           ---------------CTGTTGGCTATCACGATTCACGTCGCGGGAACGAGTCGCGTCCCG
           GGCCGCAAACGGCTACT-GGCACGAATGACGTGGCTGTCTGTGAGCCGTCTCCGTGA---
           -------CGCACGGCGGACGCTGGGTGCTGTTGCCGAAACTCGCGCTCATCACTCCGGTG
           GGCTGCGACCGCACATGC
#
#Example result for the first of 7 species: indel/deletion rate=0.2
   7    1771
A          CGTAGACGTAGCACTGTATGTTGTCC-GCGCAACCCGGGTG------GC-TGAGTTGAGC
           CCTGACC----AATCGCCCTGGCAA--------------ATGGCGCTTC-A---------
           -----------TCTGCTGAT----------GAAGGACAGCTCCG--------CAAGACCC
           CTCATAGACATGTCGCCGCG-TGCAATTAGTGACTAGC----TCGTCCCGCCCAATCTCG
           TACCTCT-------------ACGCGGCGTAGCC---------------------------
           -----CCAAA------------GACTGGTAGTGGAACTAT-------------AC----G
           GGCGATGCGCGTACGCCGAGT------------GATGGCGCCCCGGGACGTTGCCCG-GC
           TAGTGGGCGCAACGGTAGGT----------------------------------------
           -----GGAAAATACACTATTGCAGGCCTGGAATCTCTCC-------ACCGAC-------G
           TCGGCCCTGTAGCCCAGTGAAGTGACGGT----------A--------------------
           --------------GTTCATT----------TCTCCTGCCCCCC-------GG-------
           ------------------------------------------------------------
           -------------CAACGTGTCAA------------------------------------
           ---------------------GACTCGTCCTAG---------------------------
           -----CGCGGTACAGG---------------------------------------------
           -----------GGACCCCCCGG------------GT-----------CGGTAGTGGGGCG
           GTTGGTTTC------------GAGAAGTGTTCCGAAGA--------------------A-
           G---------------------------------------------------------TA
           CCGCAGC-------------------GTCCCAGCGTTAGTCGCAATGG------------
           -----------------ATGTGCCTGTCTGCATCTTACGGACAGGCCCGAGATATAGAA-
           -----CCTCCTGATC-----GTACGC--------------------------------CT
           TCGGGTTCCCATAGCCCTACCGAGTGGGTAAAGACAGTTCGGGCGTCTGGGGGGGGT---
           ACTGCTT-----------------------------------------------------
           -------------------------G----GCAAAA---GGCCGTATGGACCCGATTGTT
           GCAGAAACACTA--------GCTTGCAGACATGTAGTCGCTATCGCAGGCATAGCTGTAA
           TGTACAAATCGCACGGAGAAGTTGACG-GCGTATTC--CTCAAGGAGAATCGCGCGGTGG
           ACCTAT----------------------CCTACGCGCCGTTTGCGGTAA--TG-------
           ----------------------CCAGAGGAGG-------ATCATCAG----AGTGGAAGA
           TAAGGTTAAACCTACGTTGGAAACGGT--------------------------AGGGCC-
           ---CC----CTGGGTTGCC------------
```

FIGURE S1: Example sequences for the two simulation treatement, resulting in data that contain gaps determined by the deletion/indel parameter in the simulation software DAWG (Cartwright 2005).

2 Bird dataset

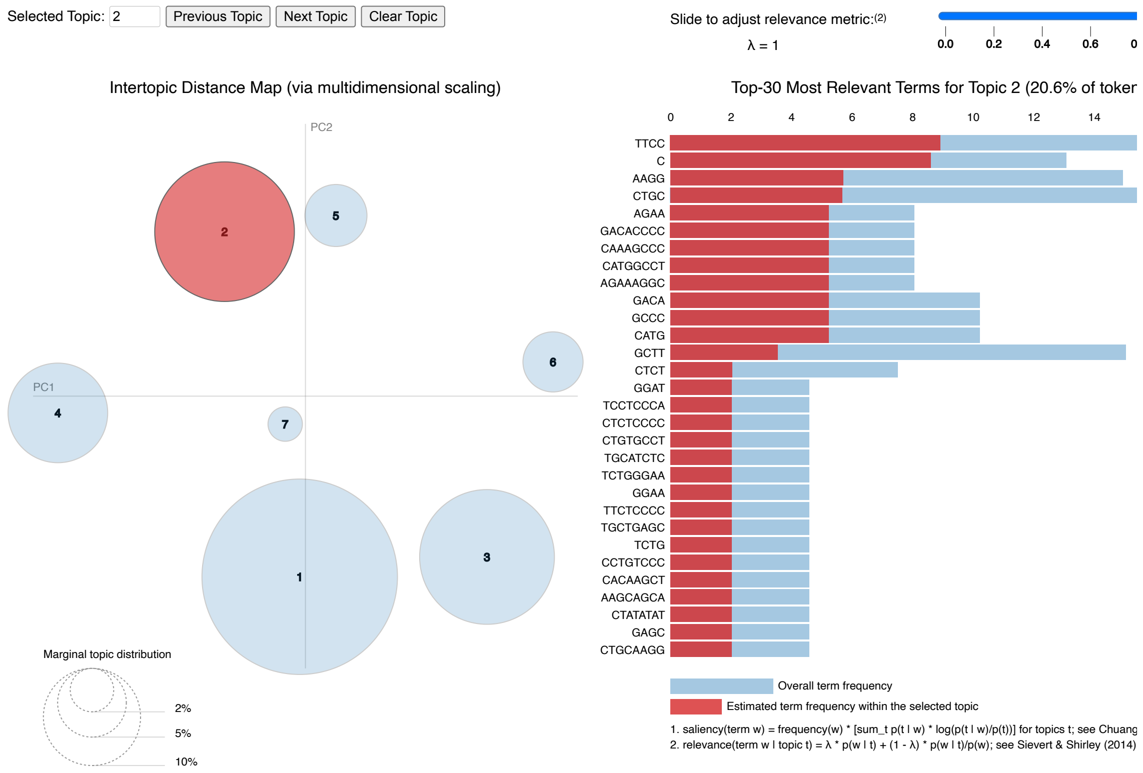

FIGURE S2: Screenshot of an interactive two-part visualization output from PYLDAVIS for the 'Bird' dataset, first locus. Left: an intertopic distance map of topics (7 topics) generated by the LDA model for the first locus. The topics are represented as circles scaled to their frequency of occurrence. The index number of each cluster represents the topic ID; the red circle shows the current topic, and the blue circles show other topics. Right: distribution of the top 30 most relevant terms among topics.

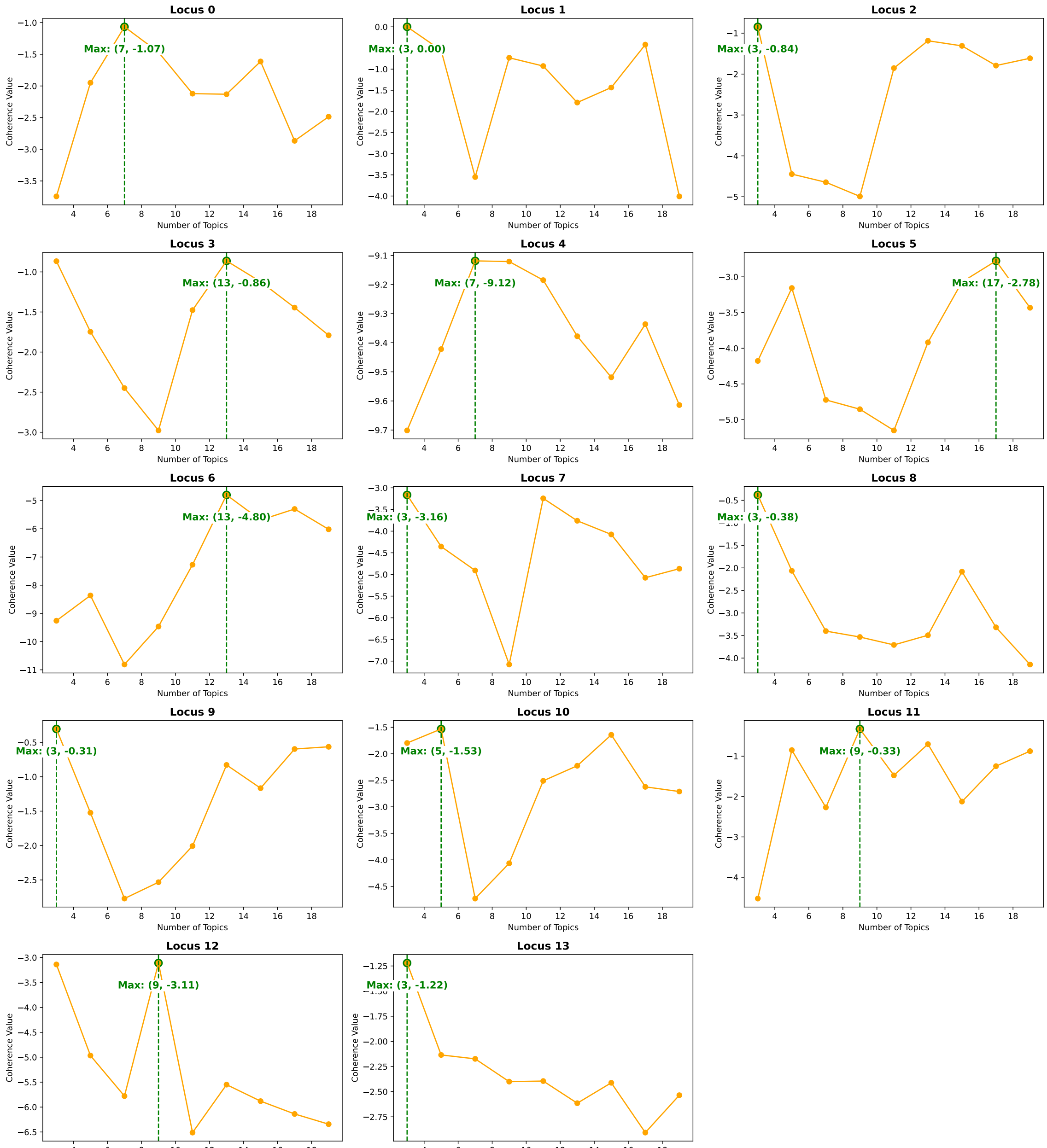

FIGURE S3: Coherence scores for varying numbers of topics in the range of 3 to 20 (with an increment of 2) for each of the 14 loci in the 'Bird' dataset. Each subplot represents the coherence analysis for a specific locus, illustrating the *u_mass* coherence scores for topic numbers 3, 5, 7, 9, 11, 13, 15, 17, and 19. Higher coherence scores indicate more interpretable topics, allowing us to determine the optimal number of topics for each locus individually.

3 Mammal dataset

Table S1: Alphabetical list of Mammal species

ailur_mel	*Ailurus fulgens* (Red Panda)
anoli_car	*Anolis carolinensis* (Carolina Anole)
balae_acu	*Balaenoptera acutorostrata* (Minke Whale)
bos_mut	*Bos mutus* (Wild Yak)
bos_tau	*Bos taurus* (Domestic Cattle)
calli_jac	*Callithrix jacchus* (Common Marmoset)
camel_fer	*Camelus ferus* (Wild Bactrian Camel)
canis_fam	*Canis familiaris* (Domestic Dog)
capra_hir	*Capra hircus* (Domestic Goat)
cavia_por	*Cavia porcellus* (Guinea Pig)
cerat_sim	*Ceratotherium simum* (White Rhinoceros)
chinc_lan	*Chinchilla lanigera* (Long-tailed Chinchilla)
chlor_sab	*Chlorocebus sabaeus* (Green Monkey)
cholo_hof	*Choloepus hoffmanni* (Hoffmann's Two-toed Sloth)
chrys_asi	*Chrysolophus amherstiae* (Lady Amherst's Pheasant)
condy_cri	*Condylura cristata* (Star-nosed Mole)
crice_gri	*Cricetulus griseus* (Chinese Hamster)
danio_rer	*Danio rerio* (Zebrafish)
dasyp_nov	*Dasypus novemcinctus* (Nine-banded Armadillo)
daube_mad	*Daubentonia madagascariensis* (Aye-aye)
dipod_ord	*Dipodomys ordii* (Ord's Kangaroo Rat)
echin_tel	*Echinops telfairi* (Small Madagascar Hedgehog)
eidol_hel	*Eidolon helvum* (African Straw-coloured Fruit Bat)
eleph_edw	*Elephas maximus* (Asian Elephant)
eptes_fus	*Eptesicus fuscus* (Big Brown Bat)
equus_cab	*Equus caballus* (Domestic Horse)
erina_eur	*Erinaceus europaeus* (European Hedgehog)
felis_cat	*Felis catus* (Domestic Cat)
galeo_var	*Galeopterus variegatus* (Flying Lemur)
gallu_gal	*Gallus gallus* (Red Junglefowl)
gaste_acu	*Gastereus aculeata* (Stickleback)
goril_gor	*Gorilla gorilla* (Western Gorilla)
heter_gla	*Heterocephalus glaber* (Naked Mole Rat)
homo_sap	*Homo sapiens* (Human)
ictid_tri	*Ictidomys tridecemlineatus* (Thirteen-lined Ground Squirrel)
jacul_jac	*Jaculus jaculus* (Lesser Egyptian Jerboa)
latim_cha	*Latimeria chalumnae* (Coelacanth)
lepto_wed	*Leptonycteris yerbabuenae* (Lesser Long-nosed Bat)
lipot_vex	*Lipotes vexillifer* (Yangtze River Dolphin)
loxod_afr	*Loxodonta africana* (African Elephant)
macac_fas	*Macaca fascicularis* (Crab-eating Macaque)
macac_mul	*Macaca mulatta* (Rhesus Macaque)
macro_eug	*Macropus eugenii* (Tammar Wallaby)
manis_pen	*Manis pentadactyla* (Chinese Pangolin)
megad_lyr	*Megaderma lyra* (Greater False Vampire Bat)
melea_gal	*Meleagris gallopavo* (Wild Turkey)
mesoc_aur	*Mesocricetus auratus* (Golden Hamster)
micro_mur	*Microtus muridae* (a species of vole)
micro_och	*Microcebus ochraceus* (Ochre Mouse Lemur)
monod_dom	*Monodelphis domestica* (Gray Short-tailed Opossum)
mus_mus	*Mus musculus* (House Mouse)
muste_put	*Mustela putorius* (European Polecat)
myoti_bra	*Myotis brandtii* (Brandt's Bat)
myoti_luc	*Myotis lucifugus* (Little Brown Bat)
nomas_leu	*Nomascus leucogenys* (Northern White-cheeked Gibbon)
ochot_pri	*Ochotona princeps* (American Pika)
octod_deg	*Octodon degus* (Degu)
odobe_ros	*Odocoileus rosenbergi* (White-tailed Deer)
orcin_orc	*Orcinus orca* (Killer Whale)
ornit_ana	*Ornithorhynchus anatinus* (Platypus)
oryct_afe	*Oryctolagus cuniculus* (European Rabbit)
oryct_cun	*Oryctolagus cuniculus* (European Rabbit)
otole_gar	*Otolemur garnettii* (Garnett's Galago)
ovis_ari	*Ovis aries* (Domestic Sheep)
pan_pan	*Pan paniscus* (Bonobo)
pan_tro	*Pan troglodytes* (Chimpanzee)
panth_hod	*Pantholops hodgsonii* (Tibetan Antelope)
panth_tig	*Panthera tigris* (Tiger)
papio_anu	*Papio anubis* (Olive Baboon)
pelod_sin	*Pelodiscus sinensis* (Chinese Softshell Turtle)
perom_man	*Peromyscus maniculatus* (Deer Mouse)
physe_mac	*Physeter macrocephalus* (Sperm whale)
pongo_abe	*Pongo abelii* (Sumatran Orangutan)
proca_cap	*Procavia capensis* (Rock Hyrax)
ptero_ale	*Pteropus alecto* (Black Flying Fox)
ptero_par	*Pteronotus parnellii* (Parnell's mustached bat)
ptero_vam	*Pteropus vampyrus* (Large Flying Fox)
rattu_nor	*Rattus norvegicus* (Norway Rat)
rhino_fer	*Rhinolophus ferrumequinum* (Greater horseshoe bat)
saimi_bol	*Saimiri boliviensis* (Bolivian Squirrel Monkey)
sarco_har	*Sarcophilus harrisii* (Tasmanian Devil)
sorex_ara	*Sorex araneus* (Eurasian Common Shrew)
sus_scr	*Sus scrofa* (Wild Boar)
tarsi_syr	*Tarsius syrichta* (Philippine Tarsier)
trich_man	*Trichechus manatus* (West Indian Manatee)
tupai_bel	*Tupaia belangeri* (Northern Treeshrew)
tupai_chi	*Tupaia chinensis* (Chinese Tree Shrew)
tursi_tru	*Tursiops truncatus* (Bottlenose Dolphin)
vicug_pac	*Vicugna pacos* (Alpaca)
xenop_tro	*Xenopus tropicalis* (Western Clawed Frog)

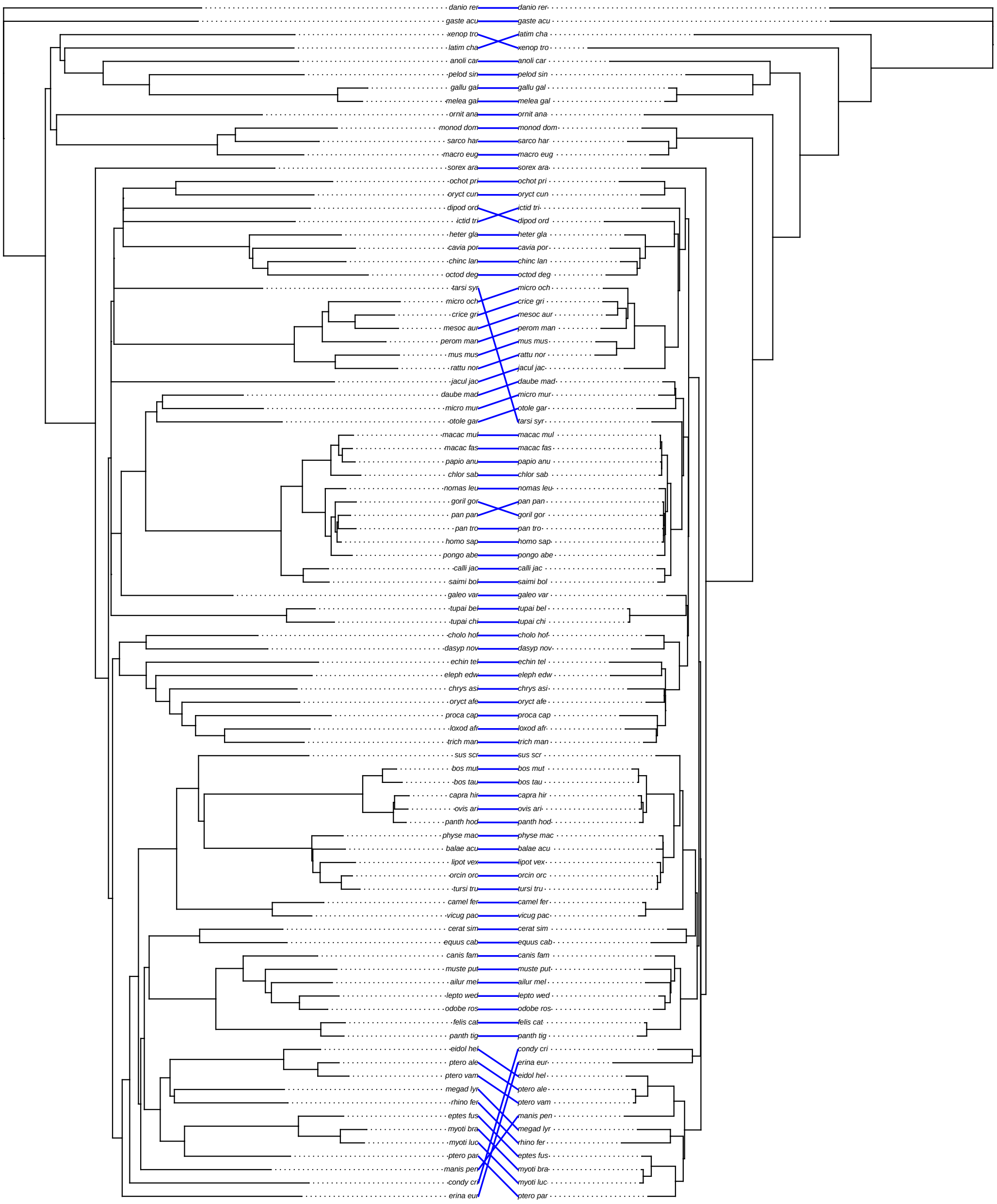

FIGURE S4: Tanglegram of the mammal dataset comparing the TOPICCONTML tree (left), generated by first removing alignment columns with gaps and then excluding k-mers containing 'N', to the maximum likelihood tree from Liu et al. (2017) (right). The alphabetical list of the species names in the tree is in Supplement Table S1.

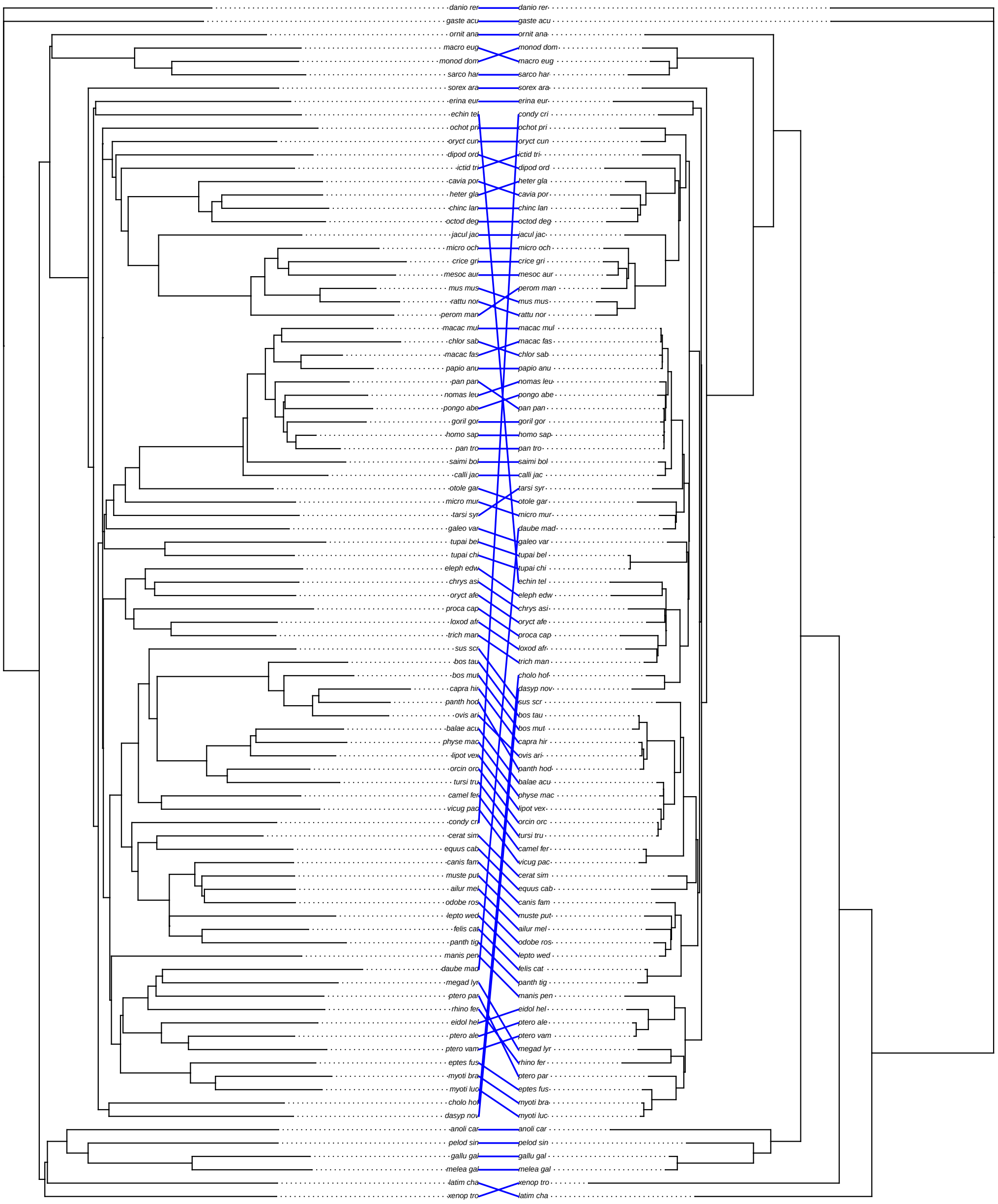

Figure S5: Tanglegram of the mammal dataset comparing the TopicContml tree (left), constructed by removing all gaps from each sequence before excluding k-mers containing 'N', to the maximum likelihood tree from Liu et al. (2017) (right). The alphabetical list of the species names in the tree is in Supplement Table S1.

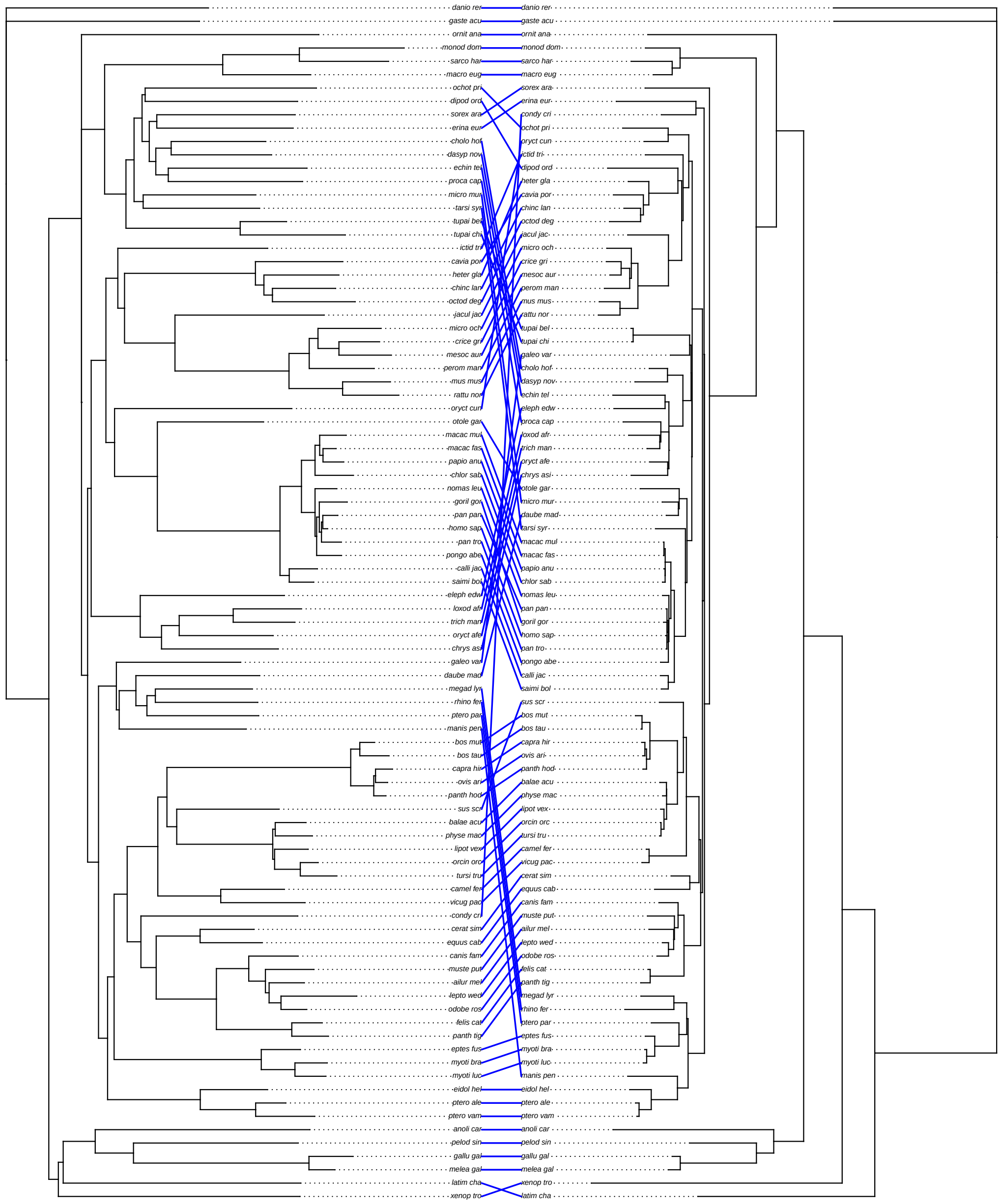

FIGURE S6: Tanglegram of the mammal dataset comparing the TOPICCONTML tree (left), constructed using aligned sequences with 'N' characters retained, to the maximum likelihood tree from Liu et al. (2017) (right). The alphabetical list of the species names in the tree is in Supplement Table S1.

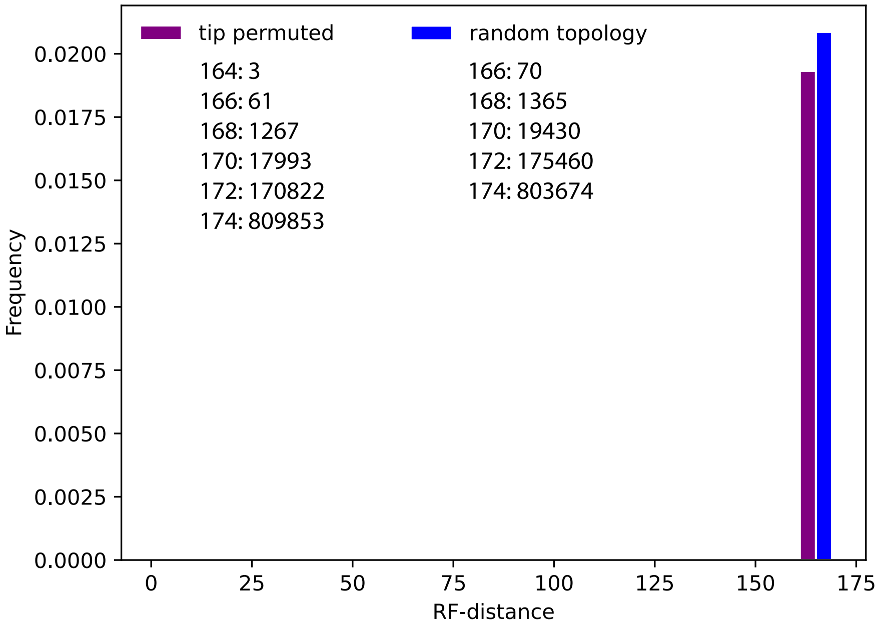

Figure S7: Robinson-Foulds (RF) distance histogram of the maximum likelihood tree of 90 species 'mammal' tree (See text) and 1,000,000 random trees with the same number of tips (random topology) and 1,000,000 trees with the true topology but with randomly permuted tip labels. The maximum likelihood tree is at 0. The inserted tables (organized as RF-steps:occurrence) give the exact distributions for each histogram. Our best TopicContml tree was 60 RF-steps from the reference tree.

4 PacBio dataset

Processing of PacBio Reads. Raw PacBio HiFi reads were downloaded in fastq.gz format from NCBI using the SRA Toolkit (https://hpc.nih.gov/apps/sratoolkit.html#doc) and from the European Nucleotide Archive for the Common Eider. To ensure uniformity of data type and quality, only PacBio HiFi reads were used. Seqkit (Shen et al. 2016) was used to obtain summary statistics for each fastq.gz file, including minimum, maximum and average read lengths. We used seqtk (https://github.com/lh3/seqtk) to subsample 100,000 or 200,000 random reads per species.

Table S2: Sources of PacBio long-read sequences from birds for TopicContml analyses.

Species	Latin name	NCBI SRA dataset no.	Average read length (bp)
Domestic chicken	Gallus gallus	SRR25731314	18,846
Wrentit	Chamaea fasciata	SRR25478075	13,086
Acorn woodpecker	Melanerpes formicivorus	SRR23445745	16,852
California quail	Callipepla californica	SRR19599932	15,661
Song sparrow	Melospiza melodia	SRR18559273	16,352
Bell's sparrow	Artemisiospiza belli	SRR18009761	17,530
Yellow warbler	Setophaga petechia	SRR20722040	17,109
Common eider	Somateria mollissima	ERR11187713	15,215
Yucatan jay	Cyanocorax yucatanicus	SRX27219773	13,003
Woodhouse's jay	Aphelocoma woodhouseii	SRX27219736	14478
Tataupa tinamou	Crypturellus tataupa	see Dryad	9,306
White-throated tinamou	Tinamus guttatus	see Dryad	7,713

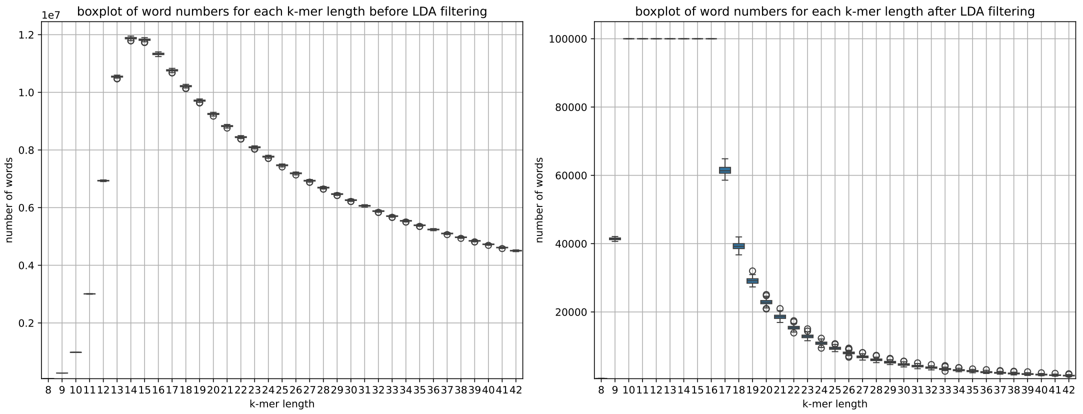

Figure S8: Comparison of word count distributions across loci and k-mer lengths ranging from 8 to 42, before and after LDA filtering for the "Unassembled Bird" dataset. The left panel depicts the variation in word numbers prior to filtering, while the right panel demonstrates the impact of LDA in refining and standardizing these distributions. Words (k-mers) appearing in fewer than 2 documents or more than 50% of documents are filtered out, retaining only the 100,000 most frequent tokens to optimize memory usage.

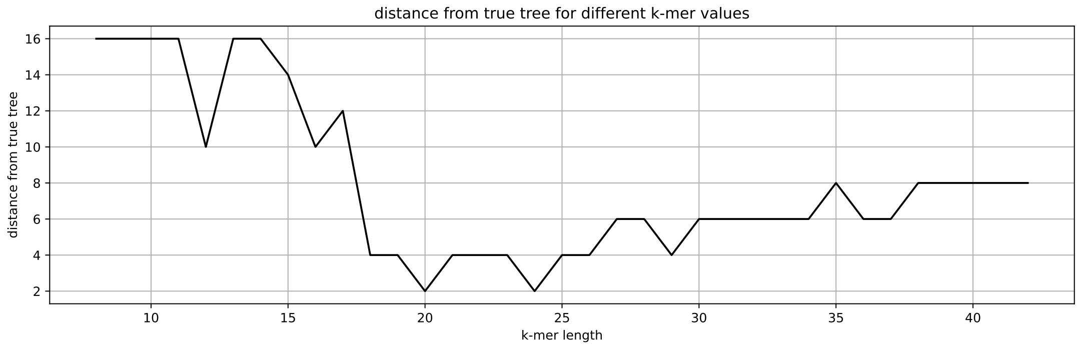

FIGURE S9: Distance between the true tree and TOPICCONTML-inferred phylogenies across varying k-mer lengths for the "Unassembled Bird" dataset. The plot shows the distances of trees generated by TOPICCONTML from the true tree, with the closest agreement observed in the 20-30 k-mer length range.

5 Runtime for different datasets

Table S3: TOPICCONTML runtime for different datasets. The first column lists the dataset names and the corresponding trees generated. The second column shows the runtime for LDA mode (if bootstrapping is applied, it shows approximate LDA time per replicate), part of phase 1. The third column displays the runtime for CONTML (if bootstrapping is applied, it shows approximate CONTML time per replicate), and the fourth column provides the total elapsed runtime of TOPICCONTML (if bootstrapping is applied, it shows the total elapsed time for all replicates).

dataset	LDA Runtime (seconds)	CONTML Runtime (seconds)	Total Runtime (seconds)
Treecreeper dataset - Bootstrap tree (1000 replicates) Figure 3b	4.25	0.06	4355.61*
Mammal dataset - Figure 4(left)	2300.63	55875.89	58288.26
PACBIO dataset - Figure 5(left)	7921.12	1.4	7922.56

* The total runtime (4355.61 seconds) for the Treecreeper dataset reflects the cumulative runtime for all 1000 bootstrap replicates.
